# The Value of Annual Glaucoma Screening for High-Risk Adults Ages 60 to 80

**DOI:** 10.7759/cureus.18710

**Published:** 2021-10-12

**Authors:** Karen Allison, Deepkumar Patel, Caren Besharim

**Affiliations:** 1 Ophthalmology, Mount Sinai Hospital, New York, USA; 2 Public Health, New York Medical College School of Health Sciences and Practice, Valhalla, USA; 3 Health policy and Management, New York Medical College School of Health Sciences and Practice, Valhalla, USA

**Keywords:** primary open angle glaucoma, genetic eye disorders screening, health policy and economics, cost effectiveness analysis, prevention of blindness

## Abstract

Glaucoma will increase in significance as a public health problem over the next three decades as the size of the aging US population grows more significant. Because glaucoma is more prevalent among African-Americans and Hispanics, and these groups will soon outnumber Caucasians. Therefore, it is even more imperative that a referral for screening protocol for high-risk groups be implemented as the standard of care. At least half of those with glaucoma do not know they have it, and the impact on the quality of life for those whose glaucoma progresses to visual impairment or blindness is significant. Without screening, glaucoma is likely to burden many families, particularly the underserved and society, unduly. Education for the public, those at increased risk, and their physicians about glaucoma, the importance of objective screening, and early treatment even for those with no symptoms will be critical toward the success of any screening protocol.

## Introduction and background

Glaucoma is a disease of the eye that deteriorates the optic nerve and leads to loss of peripheral and eventually central visual function [1]. The disease can be asymptomatic and progresses slowly. It is consistently estimated that at least half of those who have glaucoma are undiagnosed and untreated [2]. The percentage of undiagnosed and untreated is even more significant in less developed countries due to a lack of routine eye care. When left unchecked, glaucoma leads to central and peripheral vision loss and blindness. Early detection and timely treatment with medication or surgical intervention can arrest further vision loss and prevent blindness. While the destructive path of glaucoma can be halted or slowed, its deleterious effects cannot be reversed. 

Glaucoma is the leading cause of irreversible blindness, leading to blindness for 11 million people in 2020 [3]. Globally, 11% of all cases of blindness can be attributed to glaucoma [4]. An estimated 76.0 million adults worldwide, ages 40 to 80, had glaucoma in 2020, a prevalence of 3.54%. With advanced age, the likelihood of developing glaucoma increases; thus, with a rapidly ageing population, by 2040, the number of individuals with glaucoma is projected to grow to 111.8 million worldwide [5]. Primary open-angle glaucoma (POAG), which accounted for 69.2% of all glaucoma cases in 2020 and is expected to increase to 71.3% of all glaucoma cases in 2040, and primary angle-closure glaucoma (PACG), which is responsible for the remainder [5]. 

By 2050, 2 billion people globally will be age 65 or older, twice as many as in 2020 [6]. One impact of the surging senior population and its increased life expectancy is a correlated increase in morbidity from diseases such as glaucoma that affect older adults. This is especially true in Asia and Africa, where an increased lifespan is a newer phenomenon, and the birth rate has not yet experienced a corresponding slowdown. As the most densely populated continent, Asia is already home to the most significant number of people with glaucoma and this trend is expected to continue. With 60% of the global population, Asia can anticipate 42.32 million people affected by POAG in 2040 and 66.83 million overall with glaucoma in 2040, despite a prevalence of 2.31. While the population is smaller in Africa, prevalence is greater at 4.20, glaucoma is a growing concern. In Africa, the number of people with POAG is expected to nearly double from 8.73 million in 2020 to 16.26 million in 2040. Overall, glaucoma in the same period increases from 10.31 million in 2020 to 19.14 million in 2040 [5].

The most common type of glaucoma is primary open-angle glaucoma (POAG) [3]. The majority of people globally who had glaucoma in 2020, 58.6 million, had primary open-angle glaucoma. Of the 3.54% global prevalence rate, POAG accounts for 3.05; closed-angle glaucoma accounted for 0.50% of all cases [5].

 More than 90% of all glaucoma cases in the US are primary open-angle glaucoma [5]. POAG develops slowly over time, with no apparent symptoms, as intraocular pressure builds when fluids fail to drain from the eye due to blockages that have formed in its drainage canals. The open-angle refers to the angle where the iris meets the anterior sclera or peripheral cornea, and in this type of glaucoma, the angle is wide and open, as it should be in a healthy eye.

Less common in the US, closed-angle glaucoma is an acute disease of the eye caused by increased intraocular pressure due to a drainage failure. In this form of glaucoma, the iris protrudes to block drainage, constricting the iris's angle to meet the cornea. Angle-closure glaucoma is painful and characterized by sudden symptoms such as severe headache, nausea, blurry or hazy vision. Without immediate treatment, it can rapidly cause blindness. 

Prevalence in the US

In the United States, an estimated 3.36 million adults will have glaucoma in 2020. Studies indicate that more than half of all cases are undiagnosed or untreated. Glaucoma was the cause of more than 11% of all cases of blindness in the US [2]. For Blacks and Hispanics, glaucoma is the leading cause of irreversible blindness and the cause of more than one in four cases of blindness [7].

Many of those most at risk do not receive routine annual eye exams by an Eyecare professional, even when they have insurance to cover these exams. Their glaucoma then progresses untreated until it has caused irreversible - and noticeable - damage. In a 2017 screening study that targeted poor and less educated Hispanic and African-American residents of northern Manhattan, Al-Aswad and colleagues [8] found that 63% of Latinos and 55% of African-Americans in a population of 8,547 had never seen an eye doctor. Only 31.96% of those screened lacked insurance, which suggests that awareness of the importance of eye exams, the risks of glaucoma and other eye disorders, and the availability of preventive treatment is lacking among at-risk populations of low socioeconomic status. 

Older age, African or Hispanic ancestry, pronounced myopia, family history of glaucoma in first-degree relatives, hypertension, and Type 2 diabetes place an individual at increased risk for POAG [7]. High intraocular pressure, thin central cornea and corneal hysteresis, which measures the shock-absorbing ability of the cornea, are also known glaucoma risk factors for the ophthalmologist to consider during patient examination [3].

Primary open-angle glaucoma is substantially more prevalent among African-Americans and Hispanics than in Caucasians, even as prevalence increases with advancing age among all three groups. As cited by Alloco [3], Quigley estimated the prevalence among African-Americans was triple that of whites. The Eye Disease Prevalence Research Group concurred, estimating that by 2020, glaucoma would cause blindness in 50,000 African-Americans and visual impairment for another 37,000 [9].

**Figure 1:**
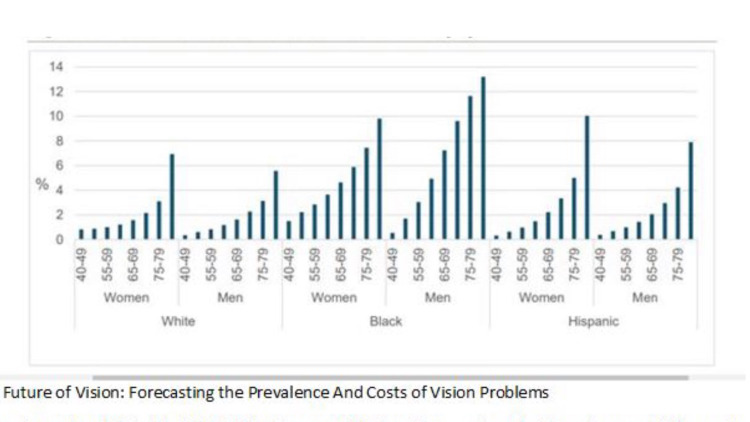
The Future of Vision: Forecasting the Prevalence And Costs of Vision Problems

**Figure 2:**
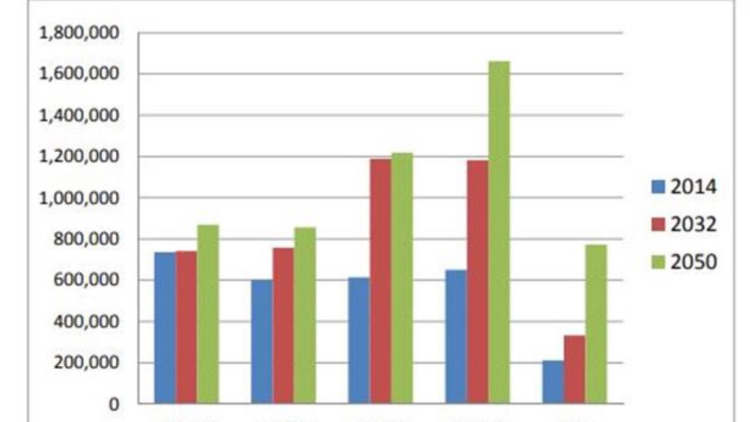
U.S. Glaucoma Population by Age Group, 2014, 2032 and 2050

**Figure 3:**
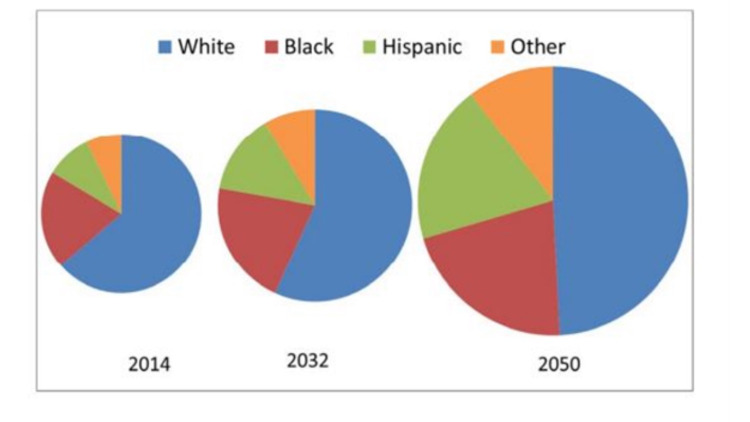
Projected U.S. Glaucoma Population by Race, 2014, 2032 and 2050

 Among those over age 75, the Salisbury Eye Evaluation Glaucoma Study reported a prevalence of glaucoma of 23.2% among Blacks, consistent with the Barbados Eye Study. Prevalence among whites over age 75 was much lower, at 9.4% among whites [10]. In their 2012 study that projected the clinical outcomes of glaucoma screening for African-Americans, Ladapo and colleagues [9] found that the prevalence of undiagnosed glaucoma among patients over age 80 was 40%. By age, prevalence is highest among adults age 65 to 80. POAG prevalence is three to four times higher among African-Americans and Hispanics than whites (Figures 1 and 2) [3]. For comparison, the prevalence per hundred women ages 70-74 was 2.16 among whites, 3.36 among Hispanics and 5.89 among Blacks. Prevalence increases markedly with age, rising to 9.4% for white ages 75 or older, while among Blacks, the prevalence was 23.2% in the same age group (Figure 3) [10].

The prevalence of open-angle glaucoma in Hispanics in their 40s is similar to whites in the same age group. However, as Hispanics age, the prevalence of OAG increases so sharply that by the time they reach their 80s, OAG prevalence in Hispanics is akin to that of Blacks. Among Hispanics age 80 and older, the prevalence was 12.02% [11]. The longitudinal Los Angeles Latino Eye Study found POAG an incidence in Hispanics at rates midway between whites and African-Americans [12].

In the Salisbury Eye Evaluation Glaucoma Study conducted in Maryland, 1 in 5 black people 73 years of age and older and 1 in 10 white people in the same age group were found to have glaucoma [10].

Ladapo found that screening patients over 80 years of age could reduce the prevalence of blindness by 10.9 % [9]. He found that earlier and more frequent screenings would have an even more significant impact. Starting when a high-risk patient was in his or her 50s, and adding glaucoma screenings at age 60 and 70, could reduce undiagnosed glaucoma by 33%, visual impairment by 6.8% and blindness by 9.9 % in African-Americans [9].

Ladapo acknowledged that his study defined visual impairment and blindness by measuring visual acuity in both eyes but failed to account for the impact on the quality of life of the loss of vision in one eye. Therefore, the benefits would be even more significant than Ladapo has presented because his method underestimated the benefits of screening and timely treatment. This study also lacked longitudinal data, which would have been helpful to capture more cases since glaucoma often progresses slowly over decades.

In The Future of Vision: Forecasting the Prevalence and Costs of Vision Problems, Wittenborn and Rein13projected that by 2050, an estimated 5.5 million people would have glaucoma. By 2050, as the tail end of the Baby Boomer generation reaches its late 80s, most glaucoma patients will be much older, with the largest patient group in the 80-89-year-old range. Slightly more than half (2.8 million vs 2.7 million) will be non-white, due to the rapid growth of the Hispanic population, with about 2.7 million white, 1.2 million black, 1.0 million Hispanic and 574,000 other minorities. This represents a population shift from majority-Caucasian to majority people of colour (Figure 1). With this shift in population, even more, people will be at risk of preventable vision loss and blindness attributable to undiagnosed and untreated glaucoma Patients must be informed about their risks, referred for appropriate and timely screenings and care and adhere to treatment regimens.

Financial burden of Glaucoma

Poor vision and blindness will burden millions more Americans over the next three decades. According to the future of vision forecast, visually impaired people are anticipated to trend upward from 3.1 million in 2014 to 5.1 million in 2032 - a 39% increase - and to 7.3 million by 2050, a 56% increase [13]. During the same period, the number of people who are blind is projected to rise from 1.4 million in 2014 to 2.2 million in 2032 and 3.1 million by 2050. 

Medical costs to treat glaucoma are expected to increase from $8.1 billion in 2021, nearly doubling to $12 billion in 2032 and to reach $17.3 billion in 2050 [13]. However, the toll of glaucoma is more than medication alone. According to Ladapo [9], Rein and colleagues said that universal glaucoma screening could save more than $10 million annually in medical costs alone if screening reduced the prevalence of vision loss by 6% to 7% in 2020. Rein and colleagues estimated that annual productivity losses and costs for nursing homes, guide dogs and other publicly provided services for patients with vision loss exceeds $11 billion. Due to the greater prevalence of POAG in African-Americans, Rein and colleagues estimated that 9% of these direct costs, or $12.2 million, were attributed to the impacts of vision loss on the African American community [14].

Medications prescribed by providers to treat glaucoma accounted for $1.2 billion in Medicare Part D prescription drug costs in 2013; those costs have indeed risen substantially since then with the increase in glaucoma cases. Glaucoma medications represent 54% of the cost of all ophthalmic drugs prescribed. The median monthly cost was $75. Glaucoma medications accounted for the greatest costs of any ophthalmic medications at $1.2 billion [15].

Medicare beneficiaries with glaucoma spent more on other health care costs besides medication. Glaucoma patients, on average, spend more on inpatient care and have more frequent home health aide visits than other Medicare beneficiaries. The majority of expenditures went toward doctor visits, inpatient care and prescriptions, with mean annual total costs of $16,760 compared to $13,094, a difference of $3,666. The mean annual costs for inpatient and outpatient care were greater for those with glaucoma than those without, with a $3,249 outpatient difference and $2,244 difference for Medicare beneficiaries with a glaucoma-related visual disability ($18,073 vs $15,829) [16].

Impact of the disease

Because the disease develops slowly, it can often go undetected until significant loss and constriction of the visual field, which can eventually lead to irreversible blindness. The worse the vision is in, the better eye, the more significant impact glaucoma has on a person [17]. However, even mild or moderate vision loss can reduce a person's productivity, restrict his social life and lead to a decline in mental health. Several studies have found that the severity of glaucoma correlates to reduced work performance that relies on eyesight, such as reading and driving [18]. As vision deteriorates, glaucoma can limit a person's independence by reducing their ability to perform activities of daily living. Poor vision can reduce work, perform housework, cook, maintain a home, or care for family members. Dependence on others to perform these activities can cause stress, anxiety and depression in the patient [19]. Declining visual acuity can discourage a person from pursuing previous passions such as reading, sewing, making crafts, engaging in a sport, or using technology. One may gradually disengage from society due to a reduced ability to recognize faces, watch television, or use a computer or smartphone. Glaucoma can make it difficult to walk on uneven ground, manoeuvre in crowds, cross the road, take the stairs or drive. Reduced central or peripheral vision can lead to reduced mobility and is widely accepted to increase the risk of falls [20].

Medicare beneficiaries, generally adults over age 65, who have glaucoma were more likely to be hospitalized and require home health aide visits than those without glaucoma [16]. Although nursing home stays were no more likely in either group. For those still employed, glaucoma can be devastating. Adaptive devices, such as talk-to-text software programs and occupational therapy, can help. Some aware of their visual loss can accommodate their activities by moving their eyes closed or turning their head. As vision deteriorates, patients may use handrails or walking sticks, add brighter lights to their homes or take public transportation when they can no longer safely drive.

Loss of vision and the attendant loss of autonomy and independence that entails can have an emotional and psychological impact leading to depression. Even at the early stages of the disease, when vision losses are more subtle, researchers found worse psychosocial functioning. People with glaucoma reported more depression, difficulty walking and falls [16]. Those with vision loss age 65 and older reported an increased prevalence of more than a dozen comorbid chronic conditions, including depression, hypertension, high cholesterol and cancer [21].

Glaucoma patients who are cognizant of the location of their visual field loss can adjust by turning their head to compensate, add brighter lights to their home and ask for help from family and friends [22].

## Review

Screening: The balance of benefits and harm

Screening for glaucoma is arduous because no single screening test is the gold standard to detect glaucoma. Instead, several tests in combination that lead to a diagnosis. These tests involve special equipment and cannot be performed in a primary care physician's office. 

Controversy continues over the effectiveness of screening interventions for the general population. In 2005, the US Preventive Services Task Force reached a neutral declaration about screening for glaucoma, stating that there was "insufficient evidence to recommend for or against screening adults for glaucoma" in the primary care setting. According to Moyer [23,24], in 2013, the US Preventive Services Task Force (USPSTF) revised its glaucoma screening recommendation to state that evidence on the accuracy of screening for POAG was limited by "the lack of an established gold standard against which individual screening tests can be compared." 

 The USPSTF found convincing evidence that treatment of increase intraocular pressure (IOP) and early glaucoma reduces the number of persons who develop small, clinically unnoticeable visual field defects and that treatment of early asymptomatic POAG decreases the number of persons whose visual field defects worsen. However, the USPSTF found no direct evidence on the benefits of screening. Suppose screening identifies glaucoma, and monitoring and treatment follows, with careful adherence. In that case, visual loss can be avoided, which appears to be a clear benefit for those most at risk of the disease.

The USPSTF noted the following: "Increased IOP, family history of glaucoma, older age, and African American race increase a person's risk for open-angle glaucoma. Recent evidence shows that glaucoma risk may be increased in Hispanics. Older African-Americans have a higher prevalence of glaucoma and perhaps a more rapid disease progression. If screening reduces vision impairment, then African-Americans would probably have greater absolute benefit than whites." [23].

This recommendation applies to adults who do not have vision symptoms and are seen in a primary care setting. The American Academy of Family Physicians supported the USPSTF recommendation and agreed that there was insufficient evidence to assess the benefits or harms of primary care screening for POAG.

 The USPSTF recommendation indicates that primary care physicians need not routinely recommend glaucoma screening for adult patients without vision symptoms. However, the statement also acknowledges that certain individuals are at increased risk for glaucoma and may benefit from screening. Although the USPSTF made no recommendation about whether primary care physicians should routinely recommend glaucoma screening for adult patients who have one or more risk factors for open-angle glaucoma, the USPSTF is currently reviewing its glaucoma screening guidelines for adults. 

 There currently is no standard of care that prompts or requires primary care physicians to refer for evaluation their patients who are in one or more categories at higher risk for glaucoma due to age or other factors. Screening can identify suspect cases of glaucoma. Referrals to an ophthalmologist can identify new cases of glaucoma. Follow up treatment can prevent further deterioration of eyesight. 

 The Centers for Medicare & Medicaid Services (CMS) provides coverage for annual glaucoma screening by ophthalmologists and optometrists for individuals in certain high-risk groups, including those with diabetes or a family history of glaucoma, African-Americans over age 50 or Hispanic-Americans 65 and older.

However, many of those most at risk do not receive routine annual eye exams by an ophthalmologist, even when they have insurance to cover these exams. Their glaucoma then progresses untreated until it has caused irreversible - and apparent - damage. In a 2017 screening study that targeted poor and less educated Hispanic and African-American residents of northern Manhattan, Al-Aswad and colleagues found that 63% of Latinos and 55% of African-Americans in a population of 8,547 had never seen an eye doctor [8]. Only 31.96% of those screened lacked insurance [8], which suggests that knowledge of the importance of eye exams and their increased risk of glaucoma lacks among at-risk populations of low socioeconomic status. 

Evidence that supports screening

**Figure 4:**
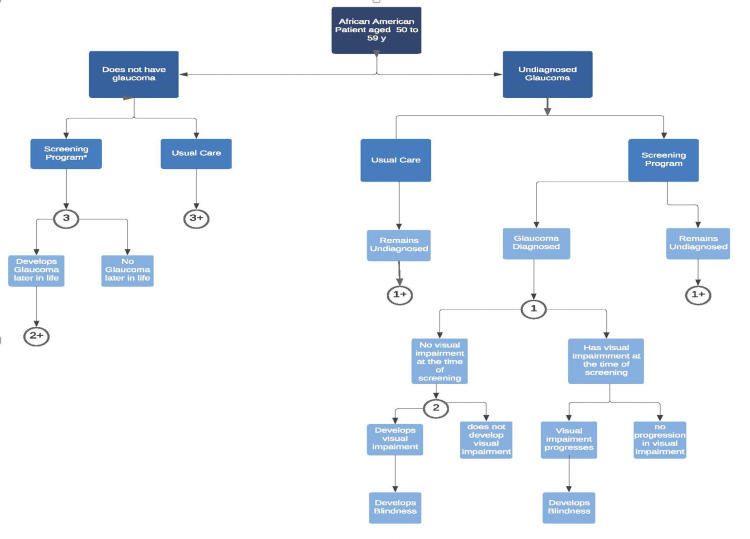
Model Screening for African-American Patient Model structure and stages of glaucoma progression in patients undergoing glaucoma screening or usual care management. Patients were African-American, between the ages of 50 and 59 years, with no history of glaucoma undergoing universal screening or usual care (sporadic screening that results in 50% of patients with glaucoma being undiagnosed). Patients with diagnosed glaucoma were treated and all patients with glaucoma were at risk of developing new or progressively worsening glaucoma-related vision loss. *Confirmatory eye examination identified patients with false-positive frequency-doubling technology results. 1,2 and 3 are probability nodes (+) represents replication of branches at node (n) where n=1,2, or 3.9

In the United States, screening studies conducted among adults age 40 and older in New York, Nogales and Tucson, AZ; Baltimore and Los Angeles offer compelling evidence that population screening of high-risk groups effectively diagnoses higher rates of glaucoma. When such screening is repeated, as indicated in Table 1 and Figure 4, followed by treatment, it can prevent blindness and visual impairment. In New York, Al-Aswad and colleagues referred 25% of those screened for confirmatory glaucoma examination [8]. As cited by Al-Aswad, Varma and colleagues found that 4.7% of those interviewed at home and examined in a clinic were diagnosed with OAG, a rate significantly higher than the 1% in whites in the Baltimore Eye Survey [12]. Similarly, Quigley and colleagues found that while glaucoma prevalence among Hispanics dovetailed with the lower rates of whites in younger age groups, it quickly surpassed white prevalence and in the oldest age groups, kept pace with the more elevated glaucoma rate of African-Americans in the Baltimore study [11]. Quigley noted that the Baltimore Eye Survey found lower socioeconomic status and education correlated with blindness from glaucoma. That study was conducted before the Affordable Care Act of 2010 when a lack of health insurance was more prevalent to avoid all but urgent medical care. However, a dearth of knowledge about the risks of glaucoma, concerns about affordability, access to preventive health services and distrust of health systems persist as barriers to care for low-income people of colour. Ladapo and colleagues [9] developed a microsimulation model to project outcomes among African-American adults who were screened for glaucoma and found that routine screening is potentially clinically effective and economical, particularly if screening is conducted frequently. He found that adults age 80 and older, who have the highest prevalence of glaucoma, would benefit the most.

**Table 1:** Evidence that support Glaucoma Screening

Publication Date	Title	Author	Study Type	Location	# of Patients	Demographics	Funding	Statistical Results
May 20, 2019, Lancet Global Health	Cost-effectiveness and cost-utility of population-based glaucoma screening in China: a decision-analytic Markov model	Tang J et al [25]	cohort	rural and urban China	Unknown	50 years, through a total of 30 1-year Markov cycles	Ulverscroft Foundation, Wenzhou Medical University Research Fund, Zheijaiang Province Health Innovation Talents Project, and Wenzhou's Ten Major Livelihood Issues 2015	Rural screening for PACG & POAG: ICER $1280 95% ci, -58-7940; ICUR $569, 95% CI 17 to 4180, screening would prevent 246 years of blindness for every 100,000 rural residents screened and 1325 years for every 100,000 urban residents screened; thus due to increased prevalence, population screening appears cost-effective
April 2018, American Journal of Ophthalmology	Improving Follow-up and Reducing Barriers for Eye Screenings in Communities: The SToP Glaucoma Study	Zhao D et al [26]	cross-sectional	Baltimore, MD	Phase 1: 686 (55%); Phase 2: 199 (63.8%); Overall: 885 (57.0%)	Age 50-plus, 91.2% African-American; 2.3% Hispanic	Centres for Disease Control and Prevention Vision Health Initiative grant	Phase 1 involved testing and refining the screening algorithm and implementing standard referral procedures; in Phase 2, the refined algorithm was adopted, screening venues were expanded; new strategies to maximize attendance at the referral exam were developed and evaluated, including providing a voucher that stated the value of the free eye exam, scheduling follow-up appointments within four weeks, showing testimonial videos on glaucoma's impact and follow-up procedure videos; Concluded that those at high risk should be made aware of the importance of screening despite a lack of symptoms
August 2017, American Journal of Ophthalmology	Optimizing Glaucoma Screening in High-Risk Population: Design and 1-Year Findings of the Screening to Prevent (SToP) Glaucoma Study	Zhao D et al [24]	prospective	Baltimore, MD	901	Age 50-plus, 94.9% African-American	Centres for Disease Control and Prevention Vision Health Initiative grant	Community-based screening intervention of underserved, high-risk population results: 39.5% referred for definitive ey exam; 43% attended the scheduled exam; 51% diagnosed with glaucoma
August 2017, Cogent Medicine	Screening for Glaucoma in Populations at High Risk: The eye Screening New York Project	Al-Aswad LA et al [8]	cross-sectional	northern Manhattan, NY	8,547	Age 20-plus, low socioeconomic status, 16.54% African-Americans, 54.37% Hispanic, 14.59% white	The Friends of the Congressional Glaucoma Caucus Foundation and Research to Prevent Blindness	25% referred for glaucoma evaluation; African-Americans and Hispanics 75% more likely than whites to need a referral
March 2012, Arch Ophthalmol 130(3): 365-372	Projected Outcomes of Glaucoma Screening in African-American Individuals	Ladapo JA et al [9]	microsimulation using data from the Eye Diseases Prevalence Research Group and Baltimore Eye Study	US	unknown	Age 50-59, African-American	Research to Prevent Blindness and National Institutes fo Health	The prevalence of undiagnosed glaucoma would be lowest at 19% among 50 and 59-year-olds and increase to 40% in those over age 80. If glaucoma screening reduced vision loss by 6% to 7% a year, it could save $10 million-plus in medical costs alone.
June 2004, Ophthalmol 111:1121-1131	The Los Angeles Latino Eye Study: design, methods and baseline data	Varma et al [12]	cross-sectional	La Puente, Los Angeles, CA	6,357	Age 40-plus, Latino, 58% female	National Eye Institute, National Center on Minority Health and the Health Disparities of the National Institutes of Health	4.7% OAG diagnosed
December 2001, Ophthalmol 119	The Prevalence of Glaucoma in a Population-Based Study of Hispanic Subjects	Quigley HA et al [11]	cross-sectional	Nogales and Tucson, AZ	4,774	Age 40-plus, Latino	Public Health Service Research, National Eye Institute, National Institutes of Health	0.50% age 41-49; 0.59% age 50-59; 1.73% age 60-69; 5.66% age 70-79; 12.02% age 80-89; 20.00 age 90 and up

It is important to note that Varma and colleagues, Zhao and colleagues and Al-Aswad and colleagues all went to considerable lengths to increase patient yield to assure robust study populations. Some screenings were conducted in community settings, and subjects' homes and clinic hours were extended to evenings and weekends to accommodate patients' work schedules. Free screenings, childcare and transportation, were provided [8,11]; field and clinic staff were Hispanic and bilingual, and outreach materials were reviewed with focus groups for cultural appropriateness [11].

Tang and colleagues analyzed the cost-effectiveness of population-based glaucoma screening in China, using a Markov model, and found that population screening for both POAG and primary angle-closure glaucoma in adults age 50 and older is likely to be cost-effective [25]. While 86% of eligible adults from rural areas and 80% of adults from urban areas participated in a community screening, follow up rates for further testing at area hospitals were expected to be as low as 19% for rural residents and 57% for city dwellers [24,25]. For this reason, annual screening was determined to be the optimal strategy. Free exams covered by national health insurance were also recommended. Tang and colleagues noted that without further education about glaucoma, neither Chinese physicians nor their patients would call for comprehensive examinations to identify this often asymptomatic disease [25].

These studies underscore the importance of physician and public education about glaucoma. Policies are needed that prompt referrals by primary-care physicians for patients in elevated risk groups and for patient support that encourages examinations, treatments and medication adherence. These may include extended hours, community screening sites, and free or low-cost screening. 

A clear and concise recommendation for primary care providers to engage in an annual glaucoma discussion once their patients reach age 60, ask a few brief screening questions and refer those at elevated risk to a screening centre and/or ophthalmologist for confirmatory testing, has the potential to prevent visual deterioration and blindness in those most at risk. By reassuring patients that Medicare and Medicaid cover such exams, primary care providers can increase the likelihood that their patients will schedule and attend such appointments for glaucoma evaluation and follow-up care. 

Referrals by primary care physicians for glaucoma screening by an ophthalmologist for a patient at age 65 and annually after that for such patients who have one or more risk factors could aid in identifying glaucoma cases earlier. As cited by Gupta and Chen, Burr and colleagues found that "high-risk screening groups increased the positive predictive value of screening tests and was shown to be cost-effective (specifically in Black patients and persons with a family history of glaucoma)." Since the presence of glaucoma in an advanced disease state is a risk factor for progression of the disease and blindness, consistent reinforcement of the importance of annual eye exams for those at risk but currently without symptoms could prevent subsequent loss of vision and blindness. Discussing the importance of treatment adherence, such as applying eye drops as prescribed, with any patients found to have glaucoma can help prevent disease progression [7].

The American Academy of Ophthalmology recommends regular eye exams for those over age 40 by an ophthalmologist or optometrist and that those with risk factors for glaucoma consider more frequent or earlier exams.

Harms related to screening

The US Preventive Services Task Force identified no direct evidence of harm caused by screening. However, it found evidence that treatment can cause several small harms, including eye irritation from medications, the risk for complications from surgery, such as the early formation of cataracts. In addition, the Task Force found there is a risk of overdiagnosis and overtreatment due to the long duration during which intraocular pressure may be high. However, there may be no detrimental impact on a patient's vision [23,24].

Cost of screening

The cost of screening is a barrier for many at higher risk despite Medicare and Medicaid coverage due to the deductible and co-payments that may be involved. Therefore, the benefit is infrequently accessed by those most at risk [26,27]. Medicare beneficiaries may pay out of pocket 20% co-payments and meet deductibles. As cited by Zhao and colleagues, Gower and colleagues found that the benefit is infrequently accessed by those most at risk [24]. Perhaps for this reason, despite their elevated risk for glaucoma, African Americans are less likely to receive routine ophthalmologic exams that detect glaucoma in its earliest stages. This means they are more likely to see a provider after they have experienced a loss of vision due to the progression of their disease. 

Studies are currently underway to assess the efficacy of community-based glaucoma screening in high-risk populations. With a cost-effective technology-based initial assessment, screening costs can be reduced, and those at greater risk are identified early and brought into care. 

Ladapo [9] considered a national, universal glaucoma screening policy for African-Americans age 50 to 59 who had never been diagnosed with glaucoma and determined that it would reduce the lifetime prevalence of undiagnosed glaucoma from 50% to 27%, with reductions in visual impairment and blindness. Ladapo estimated the costs of such screening at $80 per person, or $4,750 per new diagnosis. He calculated that it would cost $71,130 to prevent one case of visual impairment due to the need to screen 875 people and $98,970 to prevent one case of blindness due to the need to screen 1,220 people. Additional screenings at ages 60 and 70 would raise the cost of screening to $176 per person, increasing the number of people who would need to be screened to identify one case of glaucoma from 58 to 68. His costs took an automatic threshold perimetry examination and follow up eye exam with an ophthalmologist to confirm the diagnosis. Ladapo concluded that more frequent screenings would be more effective in identifying patients with glaucoma [9]. However, the initial screening should consist of intraocular pressure measurement, optic nerve evaluation, and possibly pachymetry with visual field done only if the patient shows positive findings of glaucoma. 

Targeted screening for groups at increased risk

In 2005, the United States Preventive Services Task Force found insufficient evidence to recommend for or against primary care screening of adults for glaucoma. However, the American Academy of Ophthalmology considers high-risk screening populations potentially useful and cost-effective. The Centers for Medicare & Medicaid Services provide coverage for annual screening for African-Americans over age 50, those with a family history of glaucoma and, since 2006, Hispanic-Americans age 65 and up. But as Richard K Parrish II pointed out, there is a concern that primary care providers may fail to refer patients at increased risk for glaucoma to ophthalmologists for screening due to the neutrality of the US Preventive Services Task Force on the benefits of primary care glaucoma screening for the general public [27]. Studies are currently underway to assess the efficacy of community-based glaucoma screening in high-risk populations. With a cost-effective technology-based initial assessment, screening costs can be reduced, and those at greater risk are identified early and brought into care. 

Targeted screening approach

The Screening to Prevent Glaucoma Study (SToP), based in Baltimore, focuses on African-Americans age 50 and older in a low-income neighbourhood in Baltimore, MD. Funded by the Centers for Disease Control and Prevention Vision Health Initiative, this five-year study is a pilot program whose goal is to develop a model that can be scaled nationally and be applied to other high-risk populations, such as Hispanic-Americans [26].

The SToP study has explored methods to refer those at most significant risk to develop glaucoma and reduce barriers to follow-up care. Methods used included initial screening in community settings with referrals to ophthalmologists at a nearby hospital. Investigators used text messaging and robocalls to remind patients of their appointments and schedule follow-ups. Study investigators found that even with free transportation, no out-of-pocket costs and a short distance between the screening location and the follow-up site at the hospital, just 57% of patients attended the follow-up confirmatory exam with an ophthalmologist. The study found better success, with a follow-up rate of 63.8%, when the need for the exam was explained in plain language at the initial visit, especially for asymptomatic patients. Educational videos included testimonials, provided clear information about the reason for the referral, informed patients that glaucoma can be present without symptoms, slowly robbing them of their sight, and explained the purpose of follow-up care to prevent vision loss [26]. In the group with the higher follow-up rate, patients were given vouchers that stated the dollar value of the free eye exam and appointments were scheduled within four weeks to reduce those lost to care. Among those who did present at the follow-up visit, 51% had glaucoma or were diagnosed as suspect glaucoma, demonstrating the high prevalence in this population and the importance of patient education and reducing barriers to care. 

Cost of care

Once diagnosed, glaucoma treatment is generally covered under Medicare. To reduce the intraocular pressure that leads to glaucoma-related loss of vision, individuals are typically offered medical or surgical intervention that can help them maintain their autonomy and quality of life [27,28]. Outpatient laser and incisional surgery fall under Medicare Part B. Medication coverage fall under Medicare Part D, with out-of-pocket costs varying by the plan. However, this was not always the case as Medicare Part D began to cover prescription medications on Jan. 1, 2006. After its implementation, fewer Medicare beneficiaries with glaucoma reported stretching their medication by using less or skipping doses. Glaucoma patients who reported taking smaller doses dropped from 9.4% to 2.7%, and those who skipped doses due to cost dropped from 8.2 % to 2.8%; however, the numbers of those who said they failed to fill their prescriptions remained the same [29]. Treatment adherence is critical. For some patients who do not realize the importance of consistently administering prescription eye drops to prevent further deterioration of their visual field or have difficulty paying the co-payments for these medications, their glaucoma may progress despite inconsistent treatment.

Policy recommendations

At least half of those who have POAG are unaware of their disease. POAG is estimated to go undiagnosed among 50% of all whites and blacks affected and 62% Hispanics. The undiagnosed disease becomes untreated POAG, which can progress to vision loss and blindness. Suppose annual mandatory screening referrals for those at greatest risk were implemented, with appropriate follow-up care. In that case, many with glaucoma could avoid blindness and debilitating loss of vision, with its ensuing impact on families and society.

Due to the high incidence of advanced disease in patients age 65 and older and their increased risk of vision loss and blindness, mandatory annual screening of all patients who fit one or more high-risk categories is recommended starting at age 60. Family medicine physicians, general practitioners, and internists should be provided with screening protocols to identify patients at increased risk so they can explain the importance of timely screening and be prompted to refer them to an ophthalmologist or screening centre for evaluation.

 Those at high risk include patients with Type 2 diabetes, African-American or Hispanic ancestry, or a family history of glaucoma in a first-degree relative. Additional risk factors that would be identified during screening include elevated intraocular pressure, pronounced myopia or a history of trauma.

Screening methods should include measuring intraocular pressure and fundus photography. An ophthalmologist can conduct screening at a screening centre, where technicians can perform initial screenings using non-contact fundus photography and non-contact intraocular pressure measurements, with results reviewed by an ophthalmologist. Patients will be referred for further evaluation by an ophthalmologist at various intervals depending on whether they are high, medium or low risk. Patients at low risk would be evaluated annually. Those at medium risk would be evaluated once or twice each year, while those at high risk would be evaluated more frequently.

## Conclusions

Even though progress has been made in diagnosing and treating this disease, glaucoma remains a significant burden on society. At least half of those with glaucoma do not know they have it, and the disease can progress unchecked until symptoms such as visual impairment appear. With a demographic shift to an older and more diverse US population anticipated to continue, the impact of visual impairment and blindness caused by glaucoma is expected to increase considerably in the next three decades. This will further burden a more significant number of patients, particularly the underserved. We must provide support for vision-impaired and blind residents. Educating the public, those at increased risk and their physicians about glaucoma, the importance of objective screening and early treatment even for those with no symptoms will be critical to the success of implementing a screening protocol for primary care. It also can go a long way toward encouraging those at risk to seek care, limit the progression of this disease, and lift the burden of blindness and visual impairment from millions in their golden years. 
